# Poly[bis­[μ_2_-2-(1*H*-1,2,4-triazol-1-yl)acetato]zinc(II)]

**DOI:** 10.1107/S160053680904001X

**Published:** 2009-10-07

**Authors:** Li-xia Xie, Yan Tong, Xin Li

**Affiliations:** a College of Sciences, Henan Agricultural University, Zhengzhou, Henan 450002, People’s Republic of China; b Department of Quality Examination and Management, Zhengzhou College of Animal Husbandry Engineering, Zhengzhou, Henan 450011, People’s Republic of China

## Abstract

In the title compound, [Zn(C_4_H_4_N_3_O_2_)_2_]_*n*_, the Zn^II^ atom is coordinated by two O atoms [Zn—O = 1.969 (2) and 1.997 (2) Å] and two N atoms [Zn—N = 2.046 (2) and 2.001 (2) Å] in a distorted tetra­hedral geometry. Non-classical inter­molecular C—H⋯O hydrogen bonds link the complex into a three-dimensional supra­molecular framework.

## Related literature

For related structures, see: Dixon *et al.* (2000 ▶); Fujita *et al.* (1998 ▶); Ouellette *et al.* (2006 ▶); Xie *et al.* (2009 ▶); Zhou *et al.* (2009 ▶). For the preparation of 2-(1*H*-1,2,4-triazol-1-yl)acetic acid, see: Zaderenko *et al.* (1994 ▶). 
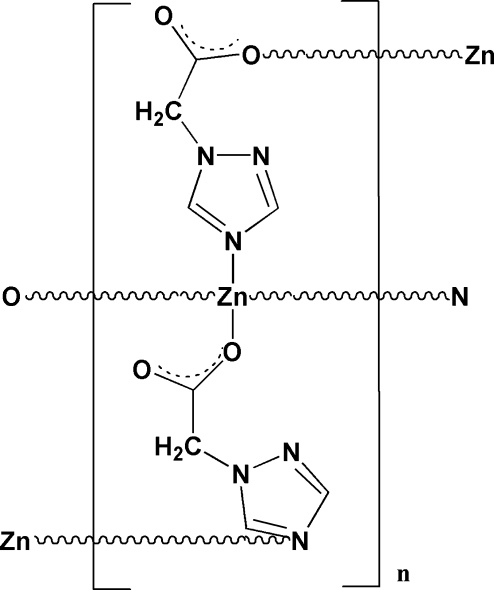


## Experimental

### 

#### Crystal data

[Zn(C_4_H_4_N_3_O_2_)_2_]
*M* *_r_* = 317.57
Monoclinic, 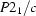

*a* = 8.791 (1) Å
*b* = 13.514 (2) Å
*c* = 10.006 (1) Å
β = 99.458 (1)°
*V* = 1172.6 (2) Å^3^
*Z* = 4
Mo *K*α radiation
μ = 2.12 mm^−1^
*T* = 293 K
0.38 × 0.20 × 0.11 mm

#### Data collection

Bruker SMART APEXII CCD diffractometer
Absorption correction: multi-scan (*SADABS*; Sheldrick, 2000 ▶) *T*_min_ = 0.609, *T*_max_ = 0.792
8509 measured reflections
2286 independent reflections
1956 reflections with *I* > 2σ(*I*)
*R*_int_ = 0.022

#### Refinement

*R*[*F* ^2^ > 2σ(*F* ^2^)] = 0.024
*wR*(*F* ^2^) = 0.057
*S* = 1.04
2286 reflections
172 parameters
H-atom parameters constrained
Δρ_max_ = 0.27 e Å^−3^
Δρ_min_ = −0.29 e Å^−3^

### 

Data collection: *APEX2* (Bruker, 2004 ▶); cell refinement: *SAINT* (Bruker, 2004 ▶); data reduction: *SAINT*; program(s) used to solve structure: *SHELXS97* (Sheldrick, 2008 ▶); program(s) used to refine structure: *SHELXL97* (Sheldrick, 2008 ▶); molecular graphics: *SHELXTL* (Sheldrick, 2008 ▶) and *DIAMOND* (Brandenburg, 1998 ▶); software used to prepare material for publication: *SHELXTL*.

## Supplementary Material

Crystal structure: contains datablocks global, I. DOI: 10.1107/S160053680904001X/lx2113sup1.cif

Structure factors: contains datablocks I. DOI: 10.1107/S160053680904001X/lx2113Isup2.hkl

Additional supplementary materials:  crystallographic information; 3D view; checkCIF report

## Figures and Tables

**Table 1:** Hydrogen-bond geometry (Å, °)

*D*—H⋯*A*	*D*—H	H⋯*A*	*D*⋯*A*	*D*—H⋯*A*
C2—H2⋯O4^i^	0.93	2.62	3.212 (3)	122
C5—H5⋯O1^ii^	0.93	2.46	3.265 (3)	145
C7—H7*A*⋯O4^iii^	0.97	2.60	3.165 (3)	118

Symmetry codes: (i) 
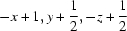
; (ii) 
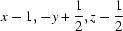
; (iii) 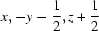.

**Fig. 1.**
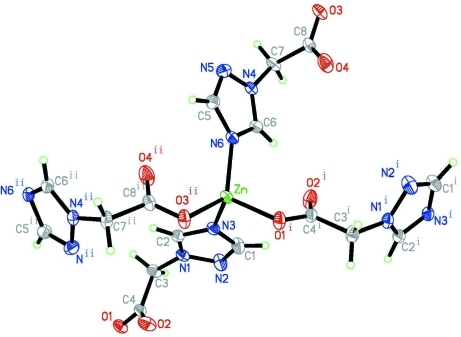
Molecular structure of the title compound, showing 30% probability displacement ellipsoids and the atom-numbering [symmetry codes: (i) - x + 2, y - 1/2, - z + 1/2; (ii) - x + 1, y + 1/2, - z + 1/2.]

**Fig. 2.**
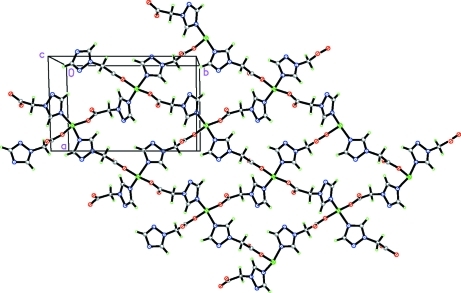
Two-dimensional polymeric structure of the title compound.

**Fig. 3.**
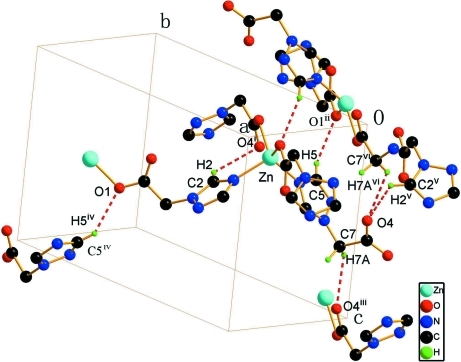
C–H···O hydrogen bonds (dotted lines) in the title compound. [symmetry codes: (i) - x + 1, y + 1/2, - z + 1/2; (ii) x - 1, - y + 1/2, z - 1/2; (iii) x, - y - 1/2, z + 1/2; (iv) x + 1, - y + 1/2, z + 1/2; (v) - x + 1, y - 1/2, - z + 1/2; (vi) x, - y - 1/2, z - 1/2.]

## supplementary crystallographic information

### Comment

The triazoles are members of the polyazaheteroaromatic class of compounds such as pyrazole, imidazole, and tetrazole, which are significant for their technological applications and for their widespread use as bridging ligands. (Ouellette *et al.*, 2006). Triazole derivatives have been studied as anti-inflammatory drug candidates and also been used as ligands for binding Pt and Ru to form antitumor metal complexes. A system takes advantage of the multifunction of the carboxylate and triazolyl group to develop the complexes (Xie *et al.*, 2009; Zhou *et al.*, 2009).

Here we report the crystal structure of the title compound (Fig. 1). Each zinc^II^ atom is four-coordinated by two nitrogen atoms (N3 and N6) and two oxygen atoms (O1 and O3) from four distinct ligands, and the coordination bond lengths of Zn–N and Zn–O are 2.046 (2), 2.001 (2) Å and 1.969 (2), 1.997 (2) Å, respectively. The coordination geometry around Zn^II^ could be described as a distorted tetrahedral configuration because the O–Zn–N coordination angles are in the range from 98.54 (6)° to 124.72 (7)° . The fully deprotonated ligand establishes a physical bridge between Zn atoms. Four Zn^II^ centers are linked together by four ligands through triazole N-donors and carboxylate O-donors into a 28-membered box macrocycle. This cavity, however, is arguably not a rectangular box(Dixon *et al.*, 2000; Fujita *et al.*, 1998), because not all the sides are truly face-to-face parallel. Taking advantage of these twists in ligands, the approximate dimensions of the rectangles are 8.5644 (9) * 8.0680 (9) Å, measured by the distance between the Zn···Zn ^i^ separations (symmetry code (i): - *x* + 2, *y* - 1/2, - *z* + 1/2), and Zn···Zn^ii^ separations (symmetry code (ii): - *x* + 1, *y* + 1/2, - *z* + 1/2). Thus the macrocycle unit is interconnected to yield a two-dimensional sheet along *ab* plane(Fig. 2). Molecules are linked by non-classical intermolecular C–H···O hydrogen bonds (Table 1 and Fig. 3).

### Experimental

The ligand 2-(1*H*-1,2,4-triazol-1-yl)acetic acid was prepared by the literature method (Zaderenko *et al.*, 1994). A solution of 2-(1*H*-1,2,4-triazol-1-yl)acetic acid (0.1 mmol), Zn(NO_3_)_2_.6H_2_O (0.1 mmol) and NaOH (0.1 mmol) in 30 ml ethanol was refluxed for 2 h, and then cooled to room temperature and filtered. After ten days, colourless single crystals suitable for X-ray analysis were obtained. Anal. Calcd(%) for C_8_H_8_ZnN_6_O_4_: C, 30.26; H, 2.54; N, 26.46. Found: C, 30.22; H, 2.60; N, 26.50.

### Refinement

All H atoms were positioned geometrically and refined using a riding model, with C–H = 0.93 Å for the triazole and 0.97 Å for the methylene H atoms. *U*_iso_(H) = 1.2*U*_eq_(C) for the triazole and the methylene H atoms.

### Crystal data

**Table:**

[Zn(C_4_H_4_N_3_O_2_)_2_]	*F*(000) = 640
*M**_r_* = 317.57	*D*_x_ = 1.799 Mg m^−^^3^
Monoclinic, *P*2_1_/*c*	Mo *K*α radiation, λ = 0.71073 Å
Hall symbol: -P 2ybc	Cell parameters from 3281 reflections
*a* = 8.791 (1) Å	θ = 2.6–26.5°
*b* = 13.514 (2) Å	µ = 2.12 mm^−^^1^
*c* = 10.006 (1) Å	*T* = 293 K
β = 99.458 (1)°	Block, colorless
*V* = 1172.6 (2) Å^3^	0.38 × 0.20 × 0.11 mm
*Z* = 4	

### Data collection

**Table:**

Bruker SMART APEXII CCD diffractometer	2286 independent reflections
Radiation source: fine-focus sealed tube	1956 reflections with *I* > 2σ(*I*)
graphite	*R*_int_ = 0.022
φ and ω scans	θ_max_ = 26.0°, θ_min_ = 2.6°
Absorption correction: multi-scan (*SADABS*; Sheldrick, 2000)	*h* = −10→10
*T*_min_ = 0.609, *T*_max_ = 0.792	*k* = −16→16
8509 measured reflections	*l* = −12→12

### Refinement

**Table:**

Refinement on *F*^2^	Primary atom site location: structure-invariant direct methods
Least-squares matrix: full	Secondary atom site location: difference Fourier map
*R*[*F*^2^ > 2σ(*F*^2^)] = 0.024	Hydrogen site location: difference Fourier map
*wR*(*F*^2^) = 0.057	H-atom parameters constrained
*S* = 1.04	*w* = 1/[σ^2^(*F*_o_^2^) + (0.0228*P*)^2^ + 0.7046*P*] where *P* = (*F*_o_^2^ + 2*F*_c_^2^)/3
2286 reflections	(Δ/σ)_max_ = 0.001
172 parameters	Δρ_max_ = 0.27 e Å^−^^3^
0 restraints	Δρ_min_ = −0.29 e Å^−^^3^

### Special details

**Table:**

Geometry. All esds (except the esd in the dihedral angle between two l.s. planes) are estimated using the full covariance matrix. The cell esds are taken into account individually in the estimation of esds in distances, angles and torsion angles; correlations between esds in cell parameters are only used when they are defined by crystal symmetry. An approximate (isotropic) treatment of cell esds is used for estimating esds involving l.s. planes.
Refinement. Refinement of F^2^ against ALL reflections. The weighted R-factor wR and goodness of fit S are based on F^2^, conventional R-factors R are based on F, with F set to zero for negative F^2^. The threshold expression of F^2^ > 2sigma(F^2^) is used only for calculating R-factors(gt) etc. and is not relevant to the choice of reflections for refinement. R-factors based on F^2^ are statistically about twice as large as those based on F, and R- factors based on ALL data will be even larger.

### Fractional atomic coordinates and isotropic or equivalent isotropic displacement parameters (Å^2^)

**Table:**

	*x*	*y*	*z*	*U*_iso_*/*U*_eq_	
Zn	0.70429 (3)	0.049976 (17)	0.14839 (2)	0.02917 (9)	
O1	1.19552 (19)	0.45868 (11)	0.46359 (16)	0.0407 (4)	
O2	1.1138 (2)	0.37002 (13)	0.27788 (17)	0.0499 (5)	
O3	0.3598 (2)	−0.34549 (11)	0.49053 (16)	0.0418 (4)	
O4	0.4781 (2)	−0.29890 (13)	0.32459 (18)	0.0585 (5)	
N1	0.9991 (2)	0.22447 (13)	0.41958 (18)	0.0335 (4)	
N2	1.0940 (2)	0.14488 (15)	0.4250 (2)	0.0470 (5)	
N3	0.8718 (2)	0.11702 (12)	0.28465 (18)	0.0321 (4)	
N4	0.4890 (2)	−0.10956 (12)	0.41086 (17)	0.0306 (4)	
N5	0.3571 (2)	−0.06552 (14)	0.3463 (2)	0.0416 (5)	
N6	0.5619 (2)	−0.01888 (12)	0.25474 (17)	0.0294 (4)	
C1	1.0113 (3)	0.08183 (18)	0.3430 (2)	0.0429 (6)	
H1	1.0456	0.0186	0.3266	0.052*	
C2	0.8698 (3)	0.20781 (16)	0.3354 (2)	0.0344 (5)	
H2	0.7893	0.2528	0.3147	0.041*	
C3	1.0519 (3)	0.31596 (17)	0.4896 (2)	0.0401 (6)	
H3A	1.1255	0.3003	0.5701	0.048*	
H3B	0.9649	0.3492	0.5181	0.048*	
C4	1.1271 (2)	0.38505 (16)	0.3997 (2)	0.0333 (5)	
C5	0.4076 (3)	−0.01127 (17)	0.2543 (2)	0.0369 (5)	
H5	0.3435	0.0289	0.1940	0.044*	
C6	0.6078 (3)	−0.08246 (16)	0.3545 (2)	0.0320 (5)	
H6	0.7085	−0.1045	0.3805	0.038*	
C7	0.4817 (3)	−0.18783 (16)	0.5093 (2)	0.0370 (5)	
H7A	0.4055	−0.1714	0.5656	0.044*	
H7B	0.5810	−0.1945	0.5674	0.044*	
C8	0.4383 (3)	−0.28544 (16)	0.4351 (2)	0.0333 (5)	

### Atomic displacement parameters (Å^2^)

**Table:**

	*U*^11^	*U*^22^	*U*^33^	*U*^12^	*U*^13^	*U*^23^
Zn	0.03301 (15)	0.02428 (14)	0.02973 (14)	−0.00137 (10)	0.00366 (10)	0.00331 (10)
O1	0.0491 (10)	0.0311 (9)	0.0414 (9)	−0.0133 (7)	0.0059 (8)	−0.0041 (7)
O2	0.0666 (12)	0.0483 (11)	0.0364 (10)	−0.0205 (9)	0.0128 (8)	−0.0035 (8)
O3	0.0579 (11)	0.0291 (8)	0.0379 (9)	−0.0138 (8)	0.0059 (8)	−0.0029 (7)
O4	0.0943 (15)	0.0394 (10)	0.0484 (11)	−0.0108 (10)	0.0309 (11)	−0.0129 (8)
N1	0.0381 (11)	0.0292 (10)	0.0333 (10)	−0.0092 (8)	0.0060 (8)	−0.0011 (8)
N2	0.0451 (13)	0.0363 (11)	0.0531 (13)	0.0013 (9)	−0.0113 (10)	−0.0015 (10)
N3	0.0319 (10)	0.0281 (9)	0.0354 (10)	−0.0043 (7)	0.0032 (8)	−0.0007 (8)
N4	0.0375 (11)	0.0251 (9)	0.0291 (9)	−0.0064 (7)	0.0054 (8)	−0.0006 (7)
N5	0.0344 (11)	0.0418 (12)	0.0494 (12)	−0.0002 (9)	0.0096 (9)	0.0028 (9)
N6	0.0322 (10)	0.0240 (9)	0.0311 (9)	−0.0026 (7)	0.0030 (8)	0.0035 (7)
C1	0.0427 (15)	0.0323 (12)	0.0493 (14)	0.0039 (10)	−0.0059 (11)	−0.0013 (11)
C2	0.0300 (12)	0.0313 (12)	0.0422 (13)	−0.0029 (9)	0.0064 (10)	−0.0008 (10)
C3	0.0503 (15)	0.0370 (13)	0.0336 (12)	−0.0152 (11)	0.0087 (11)	−0.0082 (10)
C4	0.0310 (12)	0.0282 (11)	0.0402 (13)	−0.0029 (9)	0.0044 (10)	0.0000 (9)
C5	0.0328 (13)	0.0354 (12)	0.0414 (13)	0.0032 (10)	0.0027 (10)	0.0053 (10)
C6	0.0306 (12)	0.0299 (11)	0.0347 (12)	−0.0035 (9)	0.0028 (9)	0.0027 (9)
C7	0.0543 (15)	0.0306 (12)	0.0269 (11)	−0.0139 (10)	0.0092 (10)	−0.0012 (9)
C8	0.0421 (13)	0.0273 (11)	0.0288 (11)	−0.0007 (9)	0.0003 (10)	0.0006 (9)

### Geometric parameters (Å, °)

**Table:**

Zn—O1^i^	1.9693 (15)	N4—C6	1.318 (3)
Zn—O3^ii^	1.9970 (15)	N4—N5	1.367 (3)
Zn—N6	2.0010 (17)	N4—C7	1.454 (3)
Zn—N3	2.0463 (17)	N5—C5	1.310 (3)
O1—C4	1.278 (3)	N6—C6	1.328 (3)
O1—Zn^iii^	1.9693 (15)	N6—C5	1.359 (3)
O2—C4	1.223 (3)	C1—H1	0.9300
O3—C8	1.252 (3)	C2—H2	0.9300
O3—Zn^iv^	1.9970 (15)	C3—C4	1.520 (3)
O4—C8	1.227 (3)	C3—H3A	0.9700
N1—C2	1.318 (3)	C3—H3B	0.9700
N1—N2	1.357 (3)	C5—H5	0.9300
N1—C3	1.459 (3)	C6—H6	0.9300
N2—C1	1.316 (3)	C7—C8	1.531 (3)
N3—C2	1.329 (3)	C7—H7A	0.9700
N3—C1	1.355 (3)	C7—H7B	0.9700
			
O1^i^—Zn—O3^ii^	98.54 (6)	N1—C2—H2	125.1
O1^i^—Zn—N6	112.87 (7)	N3—C2—H2	125.1
O3^ii^—Zn—N6	124.72 (7)	N1—C3—C4	111.80 (18)
O1^i^—Zn—N3	108.45 (7)	N1—C3—H3A	109.3
O3^ii^—Zn—N3	103.90 (7)	C4—C3—H3A	109.3
N6—Zn—N3	107.23 (7)	N1—C3—H3B	109.3
C4—O1—Zn^iii^	114.91 (14)	C4—C3—H3B	109.3
C8—O3—Zn^iv^	105.24 (14)	H3A—C3—H3B	107.9
C2—N1—N2	110.52 (18)	O2—C4—O1	126.0 (2)
C2—N1—C3	128.6 (2)	O2—C4—C3	120.6 (2)
N2—N1—C3	120.51 (19)	O1—C4—C3	113.40 (19)
C1—N2—N1	102.41 (18)	N5—C5—N6	114.1 (2)
C2—N3—C1	103.11 (19)	N5—C5—H5	122.9
C2—N3—Zn	127.61 (15)	N6—C5—H5	122.9
C1—N3—Zn	129.27 (15)	N4—C6—N6	109.70 (19)
C6—N4—N5	110.29 (18)	N4—C6—H6	125.2
C6—N4—C7	128.2 (2)	N6—C6—H6	125.2
N5—N4—C7	120.56 (18)	N4—C7—C8	109.48 (17)
C5—N5—N4	102.52 (18)	N4—C7—H7A	109.8
C6—N6—C5	103.36 (18)	C8—C7—H7A	109.8
C6—N6—Zn	124.11 (15)	N4—C7—H7B	109.8
C5—N6—Zn	132.40 (15)	C8—C7—H7B	109.8
N2—C1—N3	114.2 (2)	H7A—C7—H7B	108.2
N2—C1—H1	122.9	O4—C8—O3	124.2 (2)
N3—C1—H1	122.9	O4—C8—C7	118.7 (2)
N1—C2—N3	109.7 (2)	O3—C8—C7	117.06 (19)
			
C2—N1—N2—C1	−1.5 (3)	C1—N3—C2—N1	−0.7 (2)
C3—N1—N2—C1	−175.3 (2)	Zn—N3—C2—N1	−179.62 (14)
O1^i^—Zn—N3—C2	148.06 (18)	C2—N1—C3—C4	−82.7 (3)
O3^ii^—Zn—N3—C2	43.95 (19)	N2—N1—C3—C4	89.9 (2)
N6—Zn—N3—C2	−89.77 (19)	Zn^iii^—O1—C4—O2	−3.3 (3)
O1^i^—Zn—N3—C1	−30.5 (2)	Zn^iii^—O1—C4—C3	179.67 (15)
O3^ii^—Zn—N3—C1	−134.6 (2)	N1—C3—C4—O2	13.0 (3)
N6—Zn—N3—C1	91.6 (2)	N1—C3—C4—O1	−169.84 (19)
C6—N4—N5—C5	−1.4 (2)	N4—N5—C5—N6	0.9 (3)
C7—N4—N5—C5	−171.21 (18)	C6—N6—C5—N5	0.0 (3)
O1^i^—Zn—N6—C6	66.44 (18)	Zn—N6—C5—N5	−175.96 (16)
O3^ii^—Zn—N6—C6	−174.32 (15)	N5—N4—C6—N6	1.5 (2)
N3—Zn—N6—C6	−52.92 (18)	C7—N4—C6—N6	170.30 (18)
O1^i^—Zn—N6—C5	−118.3 (2)	C5—N6—C6—N4	−0.9 (2)
O3^ii^—Zn—N6—C5	0.9 (2)	Zn—N6—C6—N4	175.46 (13)
N3—Zn—N6—C5	122.3 (2)	C6—N4—C7—C8	−88.3 (3)
N1—N2—C1—N3	1.0 (3)	N5—N4—C7—C8	79.5 (2)
C2—N3—C1—N2	−0.2 (3)	Zn^iv^—O3—C8—O4	−7.6 (3)
Zn—N3—C1—N2	178.63 (16)	Zn^iv^—O3—C8—C7	170.28 (16)
N2—N1—C2—N3	1.4 (3)	N4—C7—C8—O4	30.4 (3)
C3—N1—C2—N3	174.63 (19)	N4—C7—C8—O3	−147.6 (2)

Symmetry codes: (i) −*x*+2, *y*−1/2, −*z*+1/2; (ii) −*x*+1, *y*+1/2, −*z*+1/2; (iii) −*x*+2, *y*+1/2, −*z*+1/2; (iv) −*x*+1, *y*−1/2, −*z*+1/2.

### Hydrogen-bond geometry (Å, °)

**Table:**

*D*—H···*A*	*D*—H	H···*A*	*D*···*A*	*D*—H···*A*
C2—H2···O4^ii^	0.93	2.62	3.212 (3)	122
C5—H5···O1^v^	0.93	2.46	3.265 (3)	145
C7—H7A···O4^vi^	0.97	2.60	3.165 (3)	118

Symmetry codes: (ii) −*x*+1, *y*+1/2, −*z*+1/2; (v) *x*−1, −*y*+1/2, *z*−1/2; (vi) *x*, −*y*−1/2, *z*+1/2.
